# Sex differences in comorbidities associated with Sjögren's disease

**DOI:** 10.3389/fmed.2022.958670

**Published:** 2022-08-04

**Authors:** Katelyn A. Bruno, Andrea Carolina Morales-Lara, Edsel B. Bittencourt, Habeeba Siddiqui, Gabriella Bommarito, Jenil Patel, John M. Sousou, Gary R. Salomon, Rinald Paloka, Shelby T. Watford, David O. Hodge, Scott M. Lieberman, Todd D. Rozen, Paldeep S. Atwal, Peter T. Dorsher, Lynsey A. Seim, DeLisa Fairweather

**Affiliations:** ^1^Department of Cardiovascular Medicine, Mayo Clinic, Jacksonville, FL, United States; ^2^Department of Clinical and Translational Science, Mayo Clinic, Jacksonville, FL, United States; ^3^Department of Immunology, Mayo Clinic, Jacksonville, FL, United States; ^4^Department of Physical Rehabilitation, Mayo Clinic, Jacksonville, FL, United States; ^5^Department of Quantitative Health Sciences, Mayo Clinic, Jacksonville, FL, United States; ^6^Division of Rheumatology, Allergy and Immunology, Stead Family Department of Pediatrics, Carver College of Medicine, University of Iowa, Iowa City, IA, United States; ^7^Department of Neurology, Mayo Clinic, Jacksonville, FL, United States; ^8^The Atwal Clinic, Palm Beach, FL, United States; ^9^Department of Physical Medicine and Rehabilitation, Mayo Clinic, Jacksonville, FL, United States; ^10^Department of Internal Medicine, Mayo Clinic, Jacksonville, FL, United States; ^11^Department of Environmental Health Sciences, Johns Hopkins Bloomberg School of Public Health, Baltimore, MD, United States

**Keywords:** fibromyalgia, atherosclerosis, depression, pain, migraine, hypermobile syndrome, Ehlers-Danlos syndrome, smoking

## Abstract

**Background:**

Little is known about the association of comorbidities with sex and age at diagnosis in Sjögren's disease. We tested the hypothesis that sex differences occur in comorbidities in patients with Sjögren's disease.

**Methods:**

Patients with Sjögren's disease were identified from 11/1974 to 7/2018 in the Mayo Clinic electronic medical record and assessed for 22 comorbidities according to sex and age at diagnosis.

**Results:**

Of the 13,849 patients identified with Sjögren's disease, 11,969 (86%) were women and 1,880 (14%) men, primarily white (88%) with a sex ratio of 6.4:1 women to men. The mean age at diagnosis was 57 years for women and 59.7 years for men, and 5.6% had a diagnosis of fibromyalgia at Sjögren's diagnosis. Men with Sjögren's disease were more likely than women to be a current or past smoker. The average time to diagnosis of comorbidities after diagnosis of Sjögren's disease was 2.6 years. The top comorbidities in patients with Sjögren's disease were fibromyalgia (25%), depression (21.2%) and pain (16.4%). Comorbidities that occurred more often in women were hypermobile syndromes (31:1), CREST (29:1), migraine (23:1), Ehlers-Danlos syndrome (EDS) (22:1), Raynaud's syndrome (15:1), SLE (13:1), systemic sclerosis (SSc) (13:1), and fibromyalgia (12:1). Women with Sjögren's disease were at increased risk of developing hypermobile syndromes (RR 7.27, CI 1.00–52.71, *p* = 0.05), EDS (RR 4.43, CI 1.08–18.14, *p* = 0.039), CREST (RR 4.24, CI 1.56–11.50, *p* = 0.005), migraine (RR 3.67, CI 2.39–5.62, *p* < 0.001), fibromyalgia (RR 2.26, CI 1.92–2.66, *p* < 0.001), Raynaud's syndrome (RR 2.29, CI 1.77–2.96, *p* < 0.001), SLE (RR 2.13, CI 1.64–2.76, *p* < 0.001), and SSc (RR 2.05 CI 1.44–2.92; *p* < 0.001). In contrast, men with Sjögren's were at increased risk for developing myocardial infarction (RR 0.44, CI 0.35–0.55, *p* < 0.001), atherosclerosis/CAD (RR 0.44, CI 0.39–0.49, *p* < 0.001), cardiomyopathy (RR 0.63, CI 0.46–0.86, *p* = 0.003), stroke (RR 0.66 CI 0.51–0.85, *p* = 0.001), and congestive heart failure (RR 0.70, CI 0.57–0.85, *p* < 0.001).

**Conclusions:**

The top comorbidities in Sjögren's disease were fibromyalgia, depression, and pain. Women with Sjögren's disease had a higher relative risk of developing fibromyalgia, depression, pain, migraine, hypermobile syndrome, EDS and other rheumatic autoimmune diseases. Men with Sjögren's disease had higher risk of developing cardiovascular diseases.

## Introduction

Sjögren's disease [often referred to as Sjögren's syndrome, which is a less accurate term (1)] is a chronic autoimmune disease with organ-specific and systemic features that has an estimated prevalence of 0.5–4.8%, affecting ~1.5–4 million people in the US based on a total population of 300 million (2). A study using the Rochester Epidemiology Project estimated that the age- and sex-adjusted prevalence of Sjögren's disease was 10.3 per 10,000 in 2015, with a prevalence of 16.3 per 10,000 in women and 3.1 per 10,000 in men (3). The hallmark characteristic of Sjögren's disease is diminished secretory production from the primary exocrine glands, the lacrimal (involved in tear production) and/or the salivary glands. As a result, dry eye (keratoconjunctivitis sicca) and/or dry mouth (xerostomia) are among the most commonly reported symptoms. Aside from the exocrine targets, Sjögren's disease also affects the lungs, kidneys, thyroid, muscle, skin, peripheral and central nervous system (4, 5).

Previous studies of Sjögren's disease identified multiple comorbidities but did not analyze data according to sex. Several studies found that infections (particularly oral infections) and fibromyalgia/fatigue (15–30%) occur frequently in this population (6–8). In a study of 10,414 patients with Sjögren's disease the most common comorbidities were hypertension (38%), osteoarthritis (31%), rheumatoid arthritis (RA) (18%) and systemic lupus erythematosus (SLE) (15%) (9). Another study of 1,974 patients with primary Sjögren's disease were found to be at higher risk of developing hyperlipidaemia, cardiac arrhythmias, headaches, migraines and depression (10). Comorbidities in a subsequent study of 866 patients with primary Sjögren's disease included Raynaud's syndrome (14%), Hashimoto's thyroiditis (5%) and Graves' disease (3%) (11). Joint, muscle and widespread pain characteristic of fibromyalgia are also commonly observed in patients with Sjögren's disease (12). Additionally, dry eyes and dry mouth form part of the ‘somatic symptoms' to be considered for a diagnosis of fibromyalgia according to the 2010 American College of Rheumatology diagnostic criteria (13). A meta-analysis of 14 studies found that patients with primary Sjögren's disease are at increased risk for cardiovascular morbidity, but did not analyze data according to sex (14). Thus, published studies have not determined whether sex differences exist in comorbidities or whether differences in age are present in comorbidities according to sex for patients with Sjögren's disease.

Sjögren's disease is known to occur more often in women, with some studies reporting a sex ratio as high as 16:1 women to men (15–18). More recently, the female to male ratio for Sjögren's disease has been reported to range from 6:1 in small US studies (19) to 14:1 in adults from large global studies (20, 21). Although Sjögren's disease can occur in women during child-bearing years, most cases are diagnosed soon after menopause around age 55–60 (22). Most studies examining sex differences in Sjögren's disease report differences in autoantibodies, other autoimmune diseases, fibromyalgia, lymphoma, and lung disease according to sex (15, 17, 18, 23) but have not examined whether sex and age differences occur in a large number of comorbidities. In this study we tested the hypothesis that sex differences occur in comorbidities associated with Sjögren's disease by examining 22 comorbidities (i.e., autoimmune diseases, cardiovascular diseases, chronic pain-related conditions) by sex and age at diagnosis in patients with primary and secondary Sjögren's disease from the Mayo Clinic medical record.

## Methods

### Ethics statement

Research carried out in this study was in compliance with the Helsinki Declaration. The study was approved by the Mayo Clinic Institutional Review Board and receipt of a waiver of the need to consent subjects was obtained.

### Patients

Patients with Sjögren's disease were identified from the Mayo Clinic electronic medical record (EMR) using a Mayo Clinic artificial intelligence (AI) software program (i2b2) according to International Classification of Diseases (ICD)-9 (710.2) and/or ICD-10 (M35.00, M35.01, M35.02, M35.03, M35.04, M35.09) codes from 6 November 1974 to 12 July 2018. Records were filtered for birthdays after May 1, 2004 to ensure patients were ≥18 years of age. Systemic rheumatic autoimmune diseases that often co-occur with Sjögren's disease were included as comorbidities (i.e., systemic rheumatic disease such as SLE, RA, systemic sclerosis, inflammatory myopathies) so no formal distinction was made between primary and secondary Sjögren's disease. We examined comorbidities that were present at diagnosis and comorbidities that developed after diagnosis. Retrospective data were extracted from the EMR by the Mayo Clinic Statistics Department. Demographic data included age at diagnosis, race/ethnicity, sex, vitals and 22 comorbidities.

### Statistical analysis

All statistical analyses were performed using R (version 4.0.3). Descriptive analysis was used to define the prevalence of comorbidities by sex among patients with Sjögren's disease. Fisher's exact test was performed to assess the association between sex and risk of comorbidities at diagnosis by sex (women vs. men) or age (<50 vs. ≥50) and shown as relative risk (RR) with 95% confidence intervals (CI). The risk of each type of comorbid condition after diagnosis of Sjögren's disease was estimated using the Kaplan-Meier method. The risk of each of these diagnoses between males and females was evaluated using Cox proportional hazards models. The hazard ratio (HR) and 95% CI for those estimates were provided for each condition. The purpose of the study was to examine comorbidities in relation to sex or age. Although a multivariate model could be applied to show whether an interaction exists between sex and age and comorbidities, we did not perform that analysis because we were most interested in understanding the individual relationships. A value of *p* < 0.05 was considered significant.

## Results

### Patient characteristics

Patient demographics are shown in Table 1. From the 13,849 patients with Sjögren's disease identified in the Mayo Clinic EMR, 11,969 (86%) were women and 1,880 (14%) men. The sex ratio for Sjögren's disease in this study was 6.4:1 women to men. The majority of patients with Sjögren's disease were white (88% men and women), followed by African American (women 2.7%, men 1.3%) and Asian (women 1.6%, men 1.8%). The mean age at diagnosis of patients with Sjögren's disease was 57 years for women and 59.7 years for men (*p* < 0.001), indicating that diagnosis of Sjögren's disease occurred primarily in women post-menopause and after age 50 in men in this cohort. In contrast, only around 30% of women were diagnosed with Sjögren's disease prior to age 50, and only 23% of men prior to age 50 (*p* < 0.0001). Men with Sjögren's disease were also more likely than women to be a current or past smoker (Table 2).

**Table 1:** Patient characteristics.

**Demographics**	***N***	**%**
All patients	13,849	
Women	11,969	86
Men	1,880	14
All patients with age at diagnosis	13,849	
<50 years of age at diagnosis	4,092	29
≥50 years of age at diagnosis	9,757	71
Women <50 years of age at diagnosis	3,641	30
Women ≥50 years of age at diagnosis	8,328	70
Men <50 years of age at diagnosis	451	23
Men ≥50 years of age at diagnosis	1,429	77
	**Women (*****n*** **= 11,969)**	
White	10,541	88.1
African American	324	2.7
Asian	194	1.6
American Indian/alaskan native	69	0.6
Native hawaiian/pacific islander	14	0.1
Other/unknown	827	6.9
	**Men (*****n*** **= 1,880)**	
White	1,661	88.3
African American	25	1.4
Asian	34	1.8
American Indian/alaskan native	8	0.4
Native hawaiian/pacific islander	0	0
Other/unknown	152	8.1
	**Age (years)**	***P*** **-value** ^a^
**Mean age at diagnosis**	57	
Women		
Men	59.7	*p* < 0.001

a p-value result for Fisher's exact test.

**Table 2:** More men than women with Sjögren's disease are smokers (*n* = 581).

**Sex (*n*)**	**Status**	***n***	**%**	**Difference by sex**	**Relative risk (CI)**	***P-*** **value^a^**
Women (*n* = 361)	Smoker^b^	116	32%			
	Non-smoker	245	68%			
Men (*n* = 220)	Smoker	107	49%	17%	0.67 (0.54, 0.81)	0.0001
	Non-smoker	113	51%			

a Relative risk assessed using Fisher's exact test.

b Current or past smoker.

### Sex differences in autoantibodies and DHEA

From the 13,849 patients diagnosed based on ICD-9/10 codes with Sjögren's disease in our study, we found that 45.0% tested positive for antinuclear antibodies (ANA), 51.5% positive for Sjögren's syndrome (SS)-related antigen A/Ro (SSA), 37.9% positive for SS-related antigen B/La (SSB), 38% positive for rheumatoid factor (RF), and 15.8% had low dehydroepiandrosterone (DHEA) (Table 3). A large amount of data was missing so that the number of patients examined for autoantibodies (around 2000) and DHEA (around 200) levels were far fewer than the original number of patients (around 13,000), especially for males. More women with Sjögren's disease tested positive for autoantibodies against ANA (*p* < 0.001) and SSA (*p* = 0.009) than men (Table 3). Dehydroepiandrosterone (DHEA) was low more often in men (*p* = 0.037) (Table 3), although results may change with a larger number of patients for comparison. Low DHEA has been associated with worse Sjögren's disease (17). Other autoantibodies such as SSB and rheumatoid factor were detected at similar levels in men and women (Table 3).

**Table 3:** Autoantibodies and DHEA in women with Sjögren's disease compared to men.

**Variable**	**Total (*n* = 13,849)**	**Women (*n* = 11,969)**	**Men (*n* = 1,880)**	***P*** **value^a^**
**ANA** ^b^				**<0.001**
Missing (*n*)	7,970	6,976	994	
Neg	3,235(55.0%)	2,687 (53.8%)	548 (61.9%)	
Pos	2,644 (45.0%)	2,306 (46.2%)	338 (38.1%)	
**SSA**				**0.009**
Missing (*n*)	6,680	5,740	942	
Neg	3,475(48.5%)	2,982 (47.9%)	493 (52.4%)	
Pos	3,694 (51.5%)	3,247 (52.1%)	447 (47.5%)	
SSB				0.125
Missing (*n*)	7,647	6,598	1,049	
Neg	3,851(62.1%)	3,315 (61.7%)	536 (64.5%)	
Pos	2,351 (37.9%)	2,056 (38.3%)	295 (35.5%)	
**DHEA**				**0.037**
Missing (*n*)	13,653	11,782	1,871	
High	165 (84.2%)	160 (85.6%)	5 (55.6%)	
Low to normal	31 (15.8%)	27 (14.4%)	4 (44.4%)	
RF				0.267
Missing (*n*)	8,043	7,013	1,030	
Neg	3,601 (62.0%)	3,059 (61.7%)	542 (63.8%)	
Pos	2,205 (38.0%)	1,897 (38.3%)	308 (36.2%)	

a Fisher's Exact Test for Count Data (Bold: significant p-value).

b ANA, antinuclear antibodies; DHEA, dehydroepiandrosterone; IgA, immunoglobulin A; M, men; Neg, negative; Pos, positive; RF, rheumatoid factor; SS, Sjögren's syndrome/disease; SSA/Ro, SS-related antigen A; SSB/La, SS-related antigen B.

### Age differences in autoantibodies and DHEA

We found that patients over the age of 50 with Sjögren's disease tested positive more often for SSA (*p* = 0.04) and rheumatoid factor (*p* < 0.001) and had low levels of DHEA (*p* < 0.001) compared to younger patients (Table 4). Other autoantibodies such as ANA and SSB did not differ by age (Table 4).

**Table 4:** Autoantibodies and DHEA in Sjögren's disease patients by age.

**Variable**	**Total (*n* = 13,849)**	** <50 yrs (*n* = 4,092)**	**≥50 yrs (*n* = 9,757)**	***P*** **-value^a^**
ANA^b^				0.309
Missing (*n*)	7,970	2,257	5,713	
Neg	3,235 (55.0%)	991 (54.0%)	2,244 (55.5%)	
Pos	2,644 (45.0%)	844 (46.0%)	1,800 (44.5%)	
**SSA**				**0.045**
Missing (*n*)	6,680	1,693	4,987	
Neg	3,475(48.5%)	1,203 (50.2%)	2,272 (47.6%)	
Pos	3,694 (51.5%)	1,196 (49.8%)	2,498 (52.4%)	
SSB				0.845
Missing (*n*)	7,647	2,066	5,581	
Neg	3,851 (62.1%)	1,254(61.9%)	2,597 (62.2%)	
Pos	2,351 (37.9%)	772 (38.1%)	1,579 (37.8%)	
**DHEA**				**<0.001**
Missing (*n*)	13,653	3,983	9,670	
High	165 (84.2%)	103 (94.5%)	62 (71.3%)	
Low to normal	31 (15.8%)	6 (5.5%)	25 (28.7%)	
**RF**				**<0.001**
Missing (*n*)	8,043	2,286	5,757	
Neg	3,601 (62.0%)	1,186 (65.7%)	2,415 (60.4%)	
Pos	2,205 (38.0%)	620 (34.3%)	1,585 (39.6%)	

a Fisher's Exact Test for Count Data (Bold: significant p-value).

b ANA, antinuclear antibodies; DHEA, dehydroepiandrosterone; IgA, immunoglobulin A; Neg, negative; Pos, positive; RF, rheumatoid factor; SS, Sjögren's syndrome/disease; SSA/Ro, SS-related antigen A; SSB/La, SS-related antigen B.

### Sex and age differences in autoantibodies and DHEA

The age differences found for the entire cohort were driven by women with Sjögren's disease who were more often positive for SSA (*p* = 0.055) and rheumatoid factor (*p* < 0.001) and had lower levels of DHEA (*p* < 0.001) after age 50 (Table 5), while there were no differences in the percentage of patients testing positive for autoantibodies or low DHEA in men by age (Table 6), but the number of male patients with DHEA values is too low to make conclusions.

**Table 5:** Autoantibodies and DHEA in female Sjögren's disease patients by age.

**Variable**	**W^a^ <50 (*n* = 3,641)**	**W ≥50 yrs (*n* = 8,328)**	**Total (*n* = 11,969)**	***P*** **-value^b^**
ANA				0.627
Missing (*n*)	2,028	4,948	6,976	
Neg	860 (53.3%)	1,827 (54.1%)	2,687 (53.8%)	
Pos	753 (46.7%)	1,553 (45.9%)	2,306 (46.2%)	
**SSA**				**0.055**
Missing (*n*)	1,486	4,254	5,740	
Neg	1,068 (49.6%)	1,914 (47.0%)	2,982 (47.9%)	
Pos	1,087 (50.4%)	2,160(53.0%)	3,247 (52.1%)	
SSB				0.722
Missing (*n*)	1,833	4,765	6,598	
Neg	1,122 (62.1%)	2,193 (61.5%)	3,315 (61.7%)	
Pos	686 (37.9%)	1,370 (38.5%)	2,056 (38.3%)	
**DHEA**				**<0.001**
Missing (*n*)	3,535	8,247	11,782.0	
High	101 (95.3%)	59 (72.8%)	160 (85.6%)	
Low to normal	5 (4.7%)	22 (27.2%)	27 (14.4%)	
**RF**				**<0.001**
Missing (*n*)	2,033	4,980	7,013	
Neg	1,062 (66.0%)	1,997 (59.6%)	3,059 (61.7%)	
Pos	546 (34.0%)	1,351 (40.4%)	1,897 (38.3%)	

a ANA, antinuclear antibodies; DHEA, dehydroepiandrosterone; IgA, immunoglobulin A; Neg, negative; Pos, positive; RF, rheumatoid factor; SS, Sjögren's syndrome/disease; SSA/Ro, SS-related antigen A; SSB/La, SS-related antigen B; W, women.

b Fisher's Exact Test for Count Data (Bold: significant p-value).

**Table 6:** Autoantibodies in male Sjögren's disease patients by age.

**Variable**	**M^a^ <50 (*n* = 451)**	**M ≥50 yrs (*n* = 1,429)**	**Total (*n* = 1,880)**	***P*** **-value^b^**
ANA				0.340
Missing (*n*)	229	765	994	
Neg	131 (59.0%)	417 (62.8%)	548 (61.9%)	
Pos	91 (41.0%)	247 (37.2%)	338 (38.1%)	
SSA				0.298
Missing (*n*)	207	733	940	
Neg	135 (55.3%)	358 (51.4%)	493 (52.4%)	
Pos	109 (44.7%)	338 (48.6%)	447 (47.6%)	
SSB				0.188
Missing (*n*)	233	816	1,049	
Neg	132 (60.6%)	404 (65.9%)	536 (64.5%)	
Pos	86 (39.4%)	209 (34.1%)	295 (35.5%)	
DHEA				1.000
Missing (*n*)	448	1,423	1,871	
High	2 (66.7%)	3 (50.0%)	5 (55.6%)	
Low to normal	1 (33.3%)	3 (50.0%)	4 (44.4%)	
RF				0.736
Missing (*n*)	253	777	1,030	
Neg	124 (62.6%)	418 (64.1%)	542 (63.8%)	
Pos	74(37.4%)	234 (35.9%)	308 (36.2%)	

a ANA, antinuclear antibodies; DHEA, dehydroepiandrosterone; IgA, immunoglobulin A; M, men; Neg, negative; Pos, positive; RF, rheumatoid factor; SS, Sjögren's syndrome/disease; SSA/Ro, SS-related antigen A; SSB/La, SS-related antigen B.

b Fisher's Exact Test for Count Data.

### Likelihood of having comorbidities at Sjögren's disease diagnosis by sex and age

When investigating the likelihood that a patient had a comorbidity at their diagnosis with Sjögren's disease, we found that women were more likely to have fibromyalgia at Sjögren's disease diagnosis (*p* < 0.001) (Table 7; Supplementary Table 1). The records had a lot of missing information related to comorbidities at date of diagnosis, but fibromyalgia was found to occur in 5.7% of patients diagnosed with Sjögren's disease at the time of their diagnosis, occurring more often in women with a sex ratio of 7:1 women to men (Table 7; Supplementary Table 1). When looking at the likelihood of having a comorbidity by age (using age 50 as a cut off) we found that patients in this study that were diagnosed over 50 years of age were more likely to have fibromyalgia (*p* = 0.001), pain (*p* = 0.003) and Ehlers-Danlos syndrome (EDS) (*p* = 0.007) (Table 8).

**Table 7:** Likelihood of having a comorbidity by sex at Sjögren's disease diagnosis (*n* = 13,849).

**Comorbidity**	***n***	**Diagnosis (%)**	**Sex ratio (W:M)^a^**	***P*** **-value^b^**
**Fibromyalgia**	W 682, M 95	5.7%	7:1	**<0.001**
Depression	W 454, M 52	3.8%	9:1	0.442
Pain	W 373, M 51	3.1%	7:1	0.736
Migraine	W 46, M 4	0.4%	12:1	0.149
Raynaud's syndrome	W 164, M 14	1.4%	12:1	0.409
Systemic sclerosis	W 45, M 2	0.4%	23:1	0.764
CREST	W 4, M 0	0.03%		1
Stroke	W 79, M 17	0.7%	5:1	0.885
PAH	W 118, M 20	1.0%	6:1	0.317
PH	W 105, M 20	0.9%	5:1	0.146
EDS	W 4, M 0	0.03%		1
Hypermobile	W 9, M 1	0.1%	9:1	0.29
RA	W 151, M 18	1.3%	8:1	0.788
SLE	W 144, M 13	1.2%	11:1	0.505
Polymyositis	W 19, M 0	0.2%		0.126
Dermatomyositis	W 11, M 2	0.1%	6:1	0.611
Myocarditis	W 8, M 3	0.1%	3:1	0.317
Lymphoma	W 48, M 14	0.4%	3:1	0.184
Atherosclerosis	W 235, M 81	2.0%	3:1	0.832
Myocardial infarction	W 51, M 23	0.4%	2:1	0.391
CMP	W 25, M 8	0.2%	3:1	0.645
CHF	W 100, M 29	0.8%	3:1	0.331

a CAD, coronary artery disease; CHF, congestive heart failure; CMP, cardiomyopathy; CREST, calcinosis, Raynaud's syndrome, esophageal dysmotility, sclerodactyly, and telangiectasia; EDS, Ehlers-Danlos syndrome; M, men; PAH, pulmonary arterial hypertension; PH, pulmonary hypertension; SLE, systemic lupus erythematosus; SSc, systemic sclerosis; W, women.

b Fisher's Exact Test for Count Data (Bold: significant p-value).

**Table 8:** Likelihood of having comorbidity by age at Sjögren's disease diagnosis (*n* =13,849).

**Comorbidity**	** <50 years (*n* = 4,092)**	**≥50 years (*n* = 9,757)**	**Total (*n* = 13,849)**	***P*** **-value^a^**
**Fibromyalgia**				**0.001**
Missing (*n*)	2,880	7,511	10,391	
No Dx^b^	977 (80.6%)	1,704 (75.9%)	2,681 (77.5%)	
Dx	235 (19.4%)	542 (24.1%)	777 (22.5%)	
Depression				0.710
Missing (*n*)	3,196	7,713	10,909	
No Dx	738 (82.4%)	1,696 (83.0%)	2,434 (82.8%)	
Dx	158 (17.6%)	348 (17.0%)	506 (17.2%)	
**Pain**				**0.003**
Missing (*n*)	3,319	8,255	11,574	
No Dx	655 (84.7%)	1,196 (79.6%)	1,851 (81.4%)	
-Dx	118 (15.3%)	306 (20.4%)	424 (18.6%)	
Migraine				1.000
Missing (*n*)	3,788	9,441	13,229	
No Dx	279 (91.8%)	291 (92.1%)	570 (91.9%)	
Dx	25 (8.2%)	25 (7.9%)	50 (8.1%)	
Raynaud's				1.000
Missing (*n*)	3,620	8,846	12,466	
No Dx	411 (87.1%)	794 (87.2%)	1,205 (87.1%)	
Dx	61 (12.9%)	117 (12.8%)	178 (12.9%)	
SSc^b^				0.856
Missing (*n*)	3,961	9,305	13,266	
No Dx	120 (91.6%)	416 (92.0%)	536 (91.9%)	
Dx	11 (8.4%)	36 (8.0%)	47 (8.1%)	
CREST				1.000
Missing (*n*)	4,078	9,650	13,728	
No Dx	14 (100.0%)	103 (96.3%)	117 (96.7%)	
Dx	0 (0.0%)	4 (3.7%)	4 (3.3%)	
Stroke				1.000
Missing (*n*)	4,025	9,305	13,330	
No Dx	55 (82.1%)	368 (81.4%)	423 (81.5%)	
Dx	12 (17.9%)	84 (18.6%)	96 (18.5%)	
PAH				0.684
Missing (*n*)	3,969	8,959	12,928	
No Dx	103 (83.7%)	680 (85.2%)	783 (85.0%)	
Dx	20 (16.3%)	118 (14.8%)	138 (15.0%)	
PH				1.000
Missing (*n*)	3,974	8,954	12,928	
No Dx	102 (86.4%)	694 (86.4%)	796 (86.4%)	
Dx	16 (13.6%)	109 (13.6%)	125 (13.6%)	
**EDS**				**0.007**
Missing (*n*)	4,045	9,736	13,781	
No Dx	47 (100.0%)	17(81.0%)	64 (94.1%)	
Dx	0 (0.0%)	4 (19.0%)	4 (5.9%)	
Hypermobile				0.712
Missing (*n*)	4,048	9,737	13,785	
No Dx	38 (86.4%)	16 (80.0%)	54 (84.4%)	
Dx	6 (13.6%)	4 (20.0%)	10 (15.6%)	
RA				0.103
Missing (*n*)	3,674	8,347	12,021	
No Dx	388 (92.8%)	1,271 (90.1%)	1,659 (90.8%)	
Dx	30 (7.2%)	139 (9.9%)	169 (9.2%)	
SLE				0.162
Missing (*n*)	3,614	9,033	12,647	
No Dx	424 (88.7%)	621 (85.8%)	1,045 (86.9%)	
Dx	54 (11.3%)	103 (14.2%)	157 (13.1%)	
Polymyositis				0.585
Missing (*n*)	4,055	9,668	13,723	
No Dx	33 (89.2%)	74 (83.1%)	107 (84.9%)	
Dx	4 (10.8%)	15 (16.9%)	19 (15.1%)	
Dermatomyositis				0.488
Missing (*n*)	4,074	9,707	13,781	
No Dx	16 (88.9%)	39 (78.0%)	55 (80.9%)	
Dx	2 (11.1%)	11 (22.0%)	13 (19.1%)	
Myocarditis				1.000
Missing (*n*)	4,082	9,736	13,818	
No Dx	7 (70.0%)	13 (61.9%)	20 (64.5%)	
Dx	3 (30.0%)	8 (38.1%)	11 (35.5%)	
Lymphoma				0.486
Missing (*n*)	4,021	9,474	13,495	
No Dx	61 (85.9%)	231 (81.6%)	292 (82.5%)	
Dx	10 (14.1%)	52 (18.4%)	62 (17.5%)	
Atherosclerosis				0.548
Missing (*n*)	3,952	7,887	11,839	
No Dx	121 (86.4%)	1,573 (84.1%)	1,694 (84.3%)	
Dx	19 (13.6%)	297 (15.9%)	316 (15.7%)	
Myocardial infarction				1.000
Missing (*n*)	4,046	9,317	13,363	
No Dx	39 (84.8%)	373 (84.8%)	412 (84.8%)	
Dx	7 (15.2%)	67 (15.2%)	74 (15.2%)	
Cardiomyopathy				0.378
Missing (*n*)	4,026	9,533	13,559	
No Dx	61 (92.4%)	196 (87.5%)	257 (88.6%)	
Dx	5 (7.6%)	28 (12.5%)	33 (11.4%)	
CHF				0.747
Missing (*n*)	4,010	8,988	12,998	
No Dx	71 (86.6%)	651 (84.7%)	722 (84.8%)	
Dx	11 (13.4%)	118 (15.3%)	129 (15.2%)	

a Fisher's Exact Test for Count Data (Bold: significant p-value).

b CAD, coronary artery disease; CHF, congestive heart failure; CMP, cardiomyopathy; CREST, calcinosis, Raynaud's syndrome, esophageal dysmotility, sclerodactyly, and telangiectasia; Dx, diagnosis; EDS, Ehlers-Danlos syndrome; PAH, pulmonary arterial hypertension; PH, pulmonary hypertension; SLE, systemic lupus erythematosus; SSc, systemic sclerosis.

### Average time between Sjögren's disease and comorbidity diagnosis

The mean time in years between diagnosis of Sjögren's disease and a diagnosis of 1 of 22 comorbidities including rheumatic autoimmune diseases (rheumatoid arthritis, lupus), cardiovascular diseases (myocardial infarct, congestive heart failure) or pain conditions (fibromyalgia, migraine) in women or men are found in Supplementary Table 2. The overall average time (mean) to diagnosis of a comorbidity after diagnosis of Sjögren's disease was around 2.6 years for men and women together or 2.7 years for women only, and around 2.3 years for men only.

### Comorbidities according to sex

The top comorbidities and sex ratios (women: men) in all patients with Sjögren's disease are listed in Table 9 and include fibromyalgia (24.9%, 12:1), depression (21.2%, 8:1), pain (16.4%, 8:1), atherosclerosis/ coronary artery disease (CAD) (14.5%, 3:1), rheumatoid arthritis (RA) (13.2%, 9:1), Raynaud's syndrome (10%, 15:1), and SLE (8.7%, 13:1). The comorbidities with the highest sex ratio that occurred more often in women in this study were hypermobile syndromes (31:1), CREST (29:1), migraine (23:1), EDS (22:1), Raynaud's syndrome (15:1), SLE (13:1), systemic sclerosis (SSc) (13:1), and fibromyalgia (12:1) (Table 9). All 22 of the comorbidities found in patients with Sjögren's disease in this study occurred more frequently in women than men, except for diseases that typically occur more often in men like lymphoma, myocardial infarction/CAD, congestive heart failure, cardiomyopathy and myocarditis (Table 10) (17, 24).

**Table 9:** Percentage and sex ratio of 22 comorbidities in women and men with Sjögren's disease (*n* = 13,849).

**Comorbidity**	***n***	**%**	**Sex ratio (W:M)^a^**
Fibromyalgia	W 3,190, M 268	24.9	12:1
Depression	W 2,605, M 335	21.2	8:1
Pain	W 2,013, M 262	16.4	8:1
Atherosclerosis/CAD	W 1,505, M 505	14.5	3:1
Rheumatoid arthritis	W 1,645, M 183	13.2	9:1
Raynaud's syndrome	W 1,295, M 88	10.0	15:1
SLE	W 1,117, M 85	8.7	13:1
PH	W 807, M 114	6.7	7:1
PAH	W 812, M 109	6.7	7:1
CHF	W 689, M 162	6.2	4:1
Migraine	W 595, M 26	4.5	23:1
SSc	W 542, M 41	4.2	13:1
Stroke	W 423, M 96	3.8	4:1
Myocardial infarction	W 357, M 129	3.5	3:1
Lymphoma	W 296, M 58	2.6	5:1
CMP	W 231, M 59	2.1	4:1
Polymyositis	W 112, M 14	0.9	8:1
CREST	W 117, M 4	0.9	29:1
EDS	W 65, M 3	0.5	22:1
Dermatomyositis	W 61, M 7	0.5	9:1
Hypermobile syndrome	W 62, M 2	0.5	31:1
Myocarditis	W 26, M 5	0.2	5:1

a CAD, coronary artery disease; CHF, congestive heart failure; CMP, cardiomyopathy; CREST, calcinosis, Raynaud's syndrome, esophageal dysmotility, sclerodactyly, and telangiectasia; Dx, diagnosis; EDS, Ehlers-Danlos syndrome; M, men; PAH, pulmonary arterial hypertension; PH, pulmonary hypertension; SLE, systemic lupus erythematosus; SSc, systemic sclerosis; W, women.

**Table 10:** Comorbidities in women and men with Sjögren's disease by sex (*n* = 13,849).

**Comorbidity**	**Women (*n* = 11,969)**	**Men (*n* = 1,880)**	**Total (*n* = 13,849)**	***P*** **-value^a^**
**Fibromyalgia**				**<0.001**
No Dx^b^	8,779 (73.3%)	1,612 (85.7%)	10,391 (75.0%)	
Dx	3,190 (26.7%)	268 (14.3%)	3,458 (25.0%)	
**Depression**				**<0.001**
No Dx	9,364 (78.2%)	1,545 (82.2%)	10,909 (78.8%)	
Dx	2,605 (21.8%)	335 (17.8%)	2,940 (21.2%)	
**Pain**				**0.002**
No Dx	9,956 (83.2%)	1,618 (86.1%)	11,574 (83.6%)	
Dx	2,013 (16.8%)	262 (13.9%)	2,275 (16.4%)	
**Migraine**				**<0.001**
No Dx	11,374 (95.0%)	1,854 (98.6%)	13,228 (95.5%)	
Dx	595 (5.0%)	26 (1.4%)	621 (4.5%)	
**Raynaud's syndrome**				**<0.001**
No Dx	10,674 (89.2%)	1,792 (95.3%)	12,466 (90.0%)	
Dx	1,295 (10.8%)	88 (4.7%)	1,383 (10.0%)	
**SSc**				**<0.001**
No Dx	11,427 (95.5%)	1,839 (97.8%)	13,266 (95.8%)	
Dx	542 (4.5%)	41 (2.2%)	583 (4.2%)	
**CREST**				**<0.001**
No Dx	11,852 (99.0%)	1,876 (99.8%)	13,728 (99.1%)	
Dx	117 (1.0%)	4 (0.2%)	121 (0.9%)	
**Stroke**				**0.001**
No Dx	11,546 (96.5%)	1,784 (94.9%)	13,330 (96.3%)	
Dx	423 (3.5%)	96 (5.1%)	519 (3.7%)	
PAH				0.123
No Dx	11,157 (93.2%)	1,771 (94.2%)	12,928 (93.3%)	
Dx	812 (6.8%)	109 (5.8%)	921 (6.7%)	
PH				0.296
No Dx	11,162 (93.3%)	1,766 (93.9%)	12,928 (93.3%)	
Dx	807 (6.7%)	114 (6.1%)	921 (6.7%)	
**EDS**				**0.021**
No Dx	11,904 (99.5%)	1,877 (99.8%)	13,781 (99.5%)	
Dx	65 (0.5%)	3 (0.2%)	68 (0.5%)	
**Hypermobile syndrome**				**0.010**
No Dx	11,907 (99.5%)	1,878 (99.9%)	13,785 (99.5%)	
Dx	62 (0.5%)	2 (0.1%)	64 (0.5%)	
**Rheumatoid arthritis**				**<0.001**
No Dx	10,324 (86.3%)	1,697 (90.3%)	12,021 (86.8%)	
Dx	1,645 (13.7%)	183 (9.7%)	1,828 (13.2%)	
**SLE**				**<0.001**
No Dx	10,852 (90.7%)	1,795 (95.5%)	12,647 (91.3%)	
Dx	1,117 (9.3%)	85 (4.5%)	1,202 (8.7%)	
Polymyositis				0.513
No Dx	11,857 (99.1%)	1,866 (99.3%)	13,723 (99.1%)	
Dx	112 (0.9%)	14 (0.7%)	126 (0.9%)	
Dermatomyositis				0.593
No Dx	11,908 (99.5%)	1,873 (99.6%)	13,781 (99.5%)	
Dx	61 (0.5%)	7 (0.4%)	68 (0.5%)	
Myocarditis				0.603
No Dx	11,943 (99.8%)	1,875 (99.7%)	13,818 (99.8%)	
Dx	26 (0.2%)	5 (0.3%)	31 (0.2%)	
Lymphoma				0.116
No Dx	11,673 (97.5%)	1,822 (96.9%)	13,495 (97.4%)	
Dx	296 (2.5%)	58 (3.1%)	354 (2.6%)	
**Atherosclerosis/CAD**				**<0.001**
No Dx	10,464 (87.4%)	1,375 (73.1%)	11,839 (85.5%)	
Dx	1,505 (12.6%)	505 (26.9%)	2,010 (14.5%)	
**Myocardial infarction**				**<0.001**
No Dx	11,612 (97.0%)	1,751 (93.1%)	13,363 (96.5%)	
Dx	357 (3.0%)	129 (6.9%)	486 (3.5%)	
**CMP**				**0.001**
No Dx	11,738 (98.1%)	1,821 (96.9%)	13,559 (97.9%)	
Dx	231 (1.9%)	59 (3.1%)	290 (2.1%)	
**CHF**				**<0.001**
No Dx	11,280 (94.2%)	1,718 (91.4%)	12,998 (93.9%)	
Dx	689 (5.8%)	162 (8.6%)	851 (6.1%)	

a P-values result from Fisher's test for categorical data (Bold: significant p-value).

b CAD, coronary artery disease; CHF, congestive heart failure; CMP, cardiomyopathy; CREST, calcinosis, Raynaud's syndrome, esophageal dysmotility, sclerodactyly, and telangiectasia; EDS, Ehlers-Danlos syndrome; M, men; PAH, pulmonary arterial hypertension; PH, pulmonary hypertension; SLE, systemic lupus erythematosus; SSc, systemic sclerosis; W, women.

**Figure 1:**
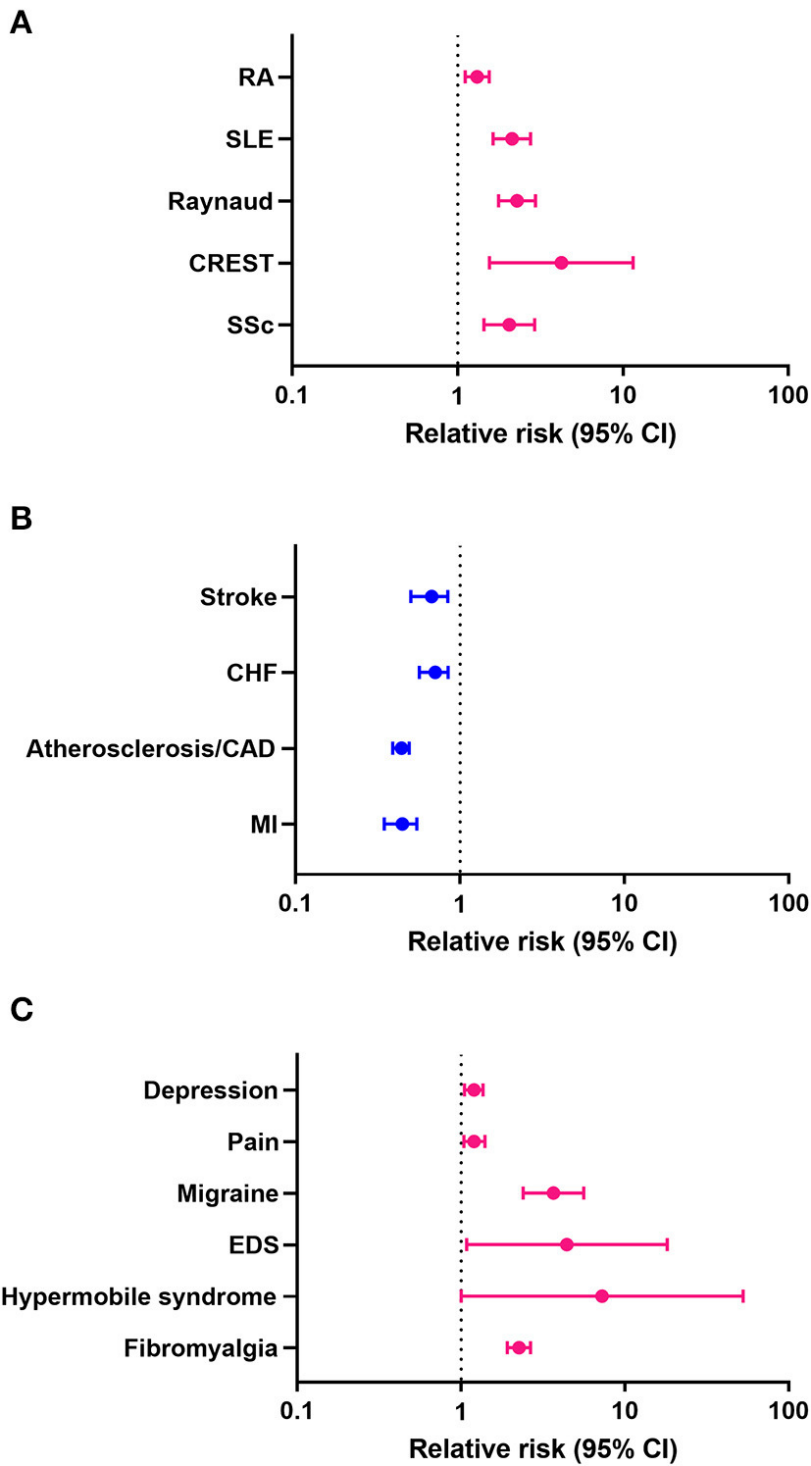
Relative risk (hazard ratio) of comorbidities in patients with Sjögren's disease (*n* = 13,849). (**A**) Rheumatic autoimmune diseases/syndromes, (**B**) cardiovascular diseases, (**C**) pain-related conditions; pink indicates increased risk in women, blue increased risk in men.

Previous studies reported that women with Sjögren's disease are at an increased risk of developing RA and SLE (9). In this study we found that women with Sjögren's disease were at greater risk of developing the rheumatic autoimmune diseases/syndromes SSc (HR 2.05, CI 1.44–2.92, *p* < 0.001), CREST (HR 4.24, CI 1.56–11.50, *p* = 0.005), Raynaud's syndrome (HR 2.29, CI 1.77–2.96, *p* < 0.001), SLE (HR 2.13, CI 1.64–2.76, *p* < 0.001), and RA (HR 1.31, CI 1.11–1.55, *p* = 0.001) than men (Table 11; Figure 1A). In contrast, men with Sjögren's disease were more likely to develop cardiovascular diseases like myocardial infarction (HR 0.44, CI 0.35–0.55, *p* < 0.001), atherosclerosis/CAD (HR 0.44, CI 0.39–0.49, *p* < 0.001), cardiomyopathy (HR 0.63, CI 0.46–0.86, *p* = 0.003), congestive heart failure (HR 0.63, CI 0.46–0.86, *p* = 0.003), and stroke (HR 0.63, CI 0.46–0.86, *p* = 0.003) (Table 11; Figure 1B). Several studies found that fibromyalgia is a leading comorbidity in Sjögren's disease (6, 7), which was confirmed in this study. Here we show that women with Sjögren's disease were at an increased risk of developing fibromyalgia (HR 2.26, CI 1.92–2.66, *p* < 0.001) (Table 11; Figure 1C). Additionally, we found that women with Sjögren's disease were more likely to have hypermobile syndromes (HR 7.27, CI 1.00–52.71, *p* = 0.05), EDS (HR 4.43, CI 1.08–18.14, *p* = 0.039), migraine (HR 3.67, CI 2.39–5.62, *p* < 0.001), pain (HR 1.20, CI 1.04–1.40, *p* = 0.014), and depression (HR 1.20, CI 1.05–1.36, *p* < 0.007) than men (Table 11; Figure 1C).

**Table 11:** Relative risk of developing a comorbidity in women vs. men with Sjögren's disease (*n* = 13,849).

**Comorbidity**	**Women**	**Men**	**HR (CI)^a^**	***P*** **-value^b^**
**Fibromyalgia**	2,140	156	**2.26 (1.92–2.66)**	<0.001
**Depression**	1,935	254	**1.20 (1.05–1.36)**	0.007
**Pain**	1,503	196	**1.20 (1.04–1.40)**	0.014
**Migraine**	513	22	**3.67 (2.39–5.62)**	<0.001
**Raynaud's syndrome**	891	62	**2.29 (1.77–2.96)**	<0.001
**SSc**	431	33	**2.05 (1.44–2.92)**	<0.001
**CREST**	110	4	**4.24 (1.56–11.50)**	0.005
Stroke	325	75	0.66 (0.51–0.85)	0.001
PAH	646	80	1.26 (1.00–1.59)	0.053
PH	648	83	1.21 (0.97–1.53)	0.096
**EDS**	56	2	**4.43 (1.08–18.14)**	0.039
**Hypermobile syndrome**	46	1	**7.27 (1.00–52.71)**	0.05
**Rheumatoid arthritis**	1,274	152	**1.31 (1.11–1.55)**	0.001
**SLE**	821	61	**2.13 (1.64–2.76)**	<0.001
Polymyositis	85	12	1.10 (0.60–2.01)	0.76
Dermatomyositis diagnosis	45	5	1.40 (0.56–3.53)	0.48
Myocarditis diagnosis	18	2	1.41 (0.33–6.08)	0.64
Lymphoma	233	43	0.83 (0.60–1.15)	0.27
Atherosclerosis/CAD	1,198	400	0.44 (0.39–0.49)	<0.001
Myocardial infarction	284	98	0.44 (0.35–0.55)	<0.001
CMP	201	49	0.63 (0.46–0.86)	0.004
CHF	557	122	0.70 (0.57–0.85)	<0.001

a CAD, coronary artery disease; CHF, congestive heart failure; CI, confidence intervals; CMP, cardiomyopathy; CREST, calcinosis, Raynaud's syndrome, esophageal dysmotility, sclerodactyly, and telangiectasia; EDS, Ehlers-Danlos syndrome; HR, hazard ratio; M, men; PAH, pulmonary arterial hypertension; PH, pulmonary hypertension; SLE, systemic lupus erythematosus; SSc, systemic sclerosis.

b Relative risk assessed using Cox Model (Bold- increased risk in women).

### Risk of developing comorbidities according to sex and age

When we examined the risk of developing comorbidities by age regardless of sex, we found that 18/22 comorbidities demonstrated age differences in the risk of developing a comorbidity with the exceptions being polymyositis, dermatomyositis, myocarditis and cardiomyopathy (Table 12). Of those comorbidities with significant differences, we found a greater risk to develop the rheumatic autoimmune diseases SSc (*p* = 0.002), CREST (*p* < 0.001) and rheumatoid arthritis (*p* < 0.001) in Sjögren's disease patients ≥50 years of age (Table 12). Also, an increased risk for many cardiovascular diseases were present after age 50 including stroke (*p* < 0.001), PAH (*p* < 0.001), PH (*p* < 0.001), atherosclerosis/ CAD (*p* < 0.001), myocardial infarct (*p* < 0.001), and congestive heart failure (*p* < 0.001) as well as lymphoma (*p* = 0.019) (Table 12). When analyzed alone, women were at increased risk of developing all of these conditions after age 50 (Tables 12, 13). In contrast, men with Sjögren's disease were not at increased risk of developing SSc, CREST or rheumatoid arthritis after age 50 (Table 14). However, they were at increased risk of developing the same cardiovascular conditions as women (Tables 12–14).

**Table 12:** Relative risk of developing a comorbidity in patients with Sjögren's disease that are ≥50 years of age (*n* = 13,849).

**Comorbidity**	** <50 yr^a^ (*n*)**	**≥50 yr (*n*)**	**HR (CI)**	***P*** **-value^b^**
Fibromyalgia	837	1,459	0.65 (0.60–0.71)	<0.001
Depression	678	1,511	0.86 (0.78–0.94)	<0.001
Pain	602	1,097	0.69 (0.63–0.77)	<0.001
Migraine	264	271	0.39 (0.33–0.46)	<0.001
Raynaud's syndrome	346	607	0.68 (0.59–0.77)	<0.001
**SSc^c^**	100	364	**1.43 (1.15–1.79)**	0.002
**CREST**	13	101	**3.23 (1.81–5.76)**	<0.001
**Stroke**	54	346	**2.59 (1.94–3.45)**	<0.001
**PAH**	101	625	**2.52 (2.04–3.11)**	<0.001
**PH**	99	632	**2.61 (2.11–3.22)**	<0.001
EDS	44	14	0.12 (0.07–0.22)	<0.001
Hypermobile syndrome	33	14	0.16 (0.09–0.31)	<0.001
**Rheumatoid arthritis**	336	1,090	**1.30 (1.15–1.47)**	<0.001
SLE	363	519	0.55 (0.48–0.63)	<0.001
Polymyositis	31	66	0.84 (0.55–1.30)	0.44
Dermatomyositis Diag	14	36	1.00 (0.54–1.85)	0.99
Myocarditis diagnosis	7	13	0.71 (0.28–1.78)	0.46
**Lymphoma**	61	215	**1.41 (1.06–1.87)**	0.019
**Atherosclerosis/CAD**	118	1,480	**5.35 (4.43–6.46)**	<0.001
**Myocardial infarction**	37	345	**3.78 (2.69–5.31)**	<0.001
CMP	59	191	1.30 (0.97–1.75)	0.077
**CHF**	67	611	**3.69 (2.87–4.76)**	<0.001

a CAD, coronary artery disease; CHF, congestive heart failure; CI, confidence interval; CMP, cardiomyopathy; CREST, calcinosis, Raynaud's syndrome, esophageal dysmotility, sclerodactyly, and telangiectasia; EDS, Ehlers-Danlos syndrome; M, men; PAH, pulmonary arterial hypertension; PH, pulmonary hypertension; RR, relative risk; SLE, systemic lupus erythematosus; SSc, systemic sclerosis; yr, year.

b Relative risk assessed using Cox Model.

c Bold- increased risk in patients ≥50 years of age.

**Table 13:** Relative risk of developing a comorbidity in women with Sjögren's disease ≥50 years (*n* = 11,969).

**Comorbidity**	** <50 yr^a^**	**≥50 yr**	**HR (CI)**	***P*** **-value^b^**
Fibromyalgia	788	1,352	0.67 (0.61–0.73)	<0.001
Depression	611	1,324	0.86 (0.78–0.95)	0.002
Pain	547	956	0.69 (0.62–0.76)	<0.001
Migraine	256	257	0.39 (0.33–0.46)	<0.001
Raynaud's syndrome	327	564	0.69 (0.60–0.79)	<0.001
**SSc^c^**	95	336	**1.44 (1.15–1.81)**	0.002
**CREST**	12	98	**3.49 (1.91–6.36)**	<0.001
**Stroke**	46	279	**2.53 (1.85–3.46)**	<0.001
**PAH**	95	551	**2.45 (1.97–3.05)**	<0.001
**PH**	91	557	**2.59 (2.07–3.24)**	<0.001
EDS	42	14	0.13 (0.07–0.24)	<0.001
Hypermobile syndrome	33	13	0.16 (0.08–0.30)	<0.001
**Rheumatoid arthritis**	310	964	**1.29 (1.14–1.47)**	<0.001
SLE	347	474	0.54 (0.47–0.63)	<0.001
Polymyositis	26	59	0.94 (0.59–1.50)	0.8
Dermatomyositis diag	11	34	1.25 (0.63–2.46)	0.52
Myocarditis diagnosis	7	11	0.62 (0.24–1.61)	0.33
**Lymphoma**	49	184	**1.56 (1.14–2.14)**	0.006
**Atherosclerosis/CAD**	90	1,108	**5.43 (4.38–6.74)**	<0.001
**Myocardial infarction**	30	254	**3.51 (2.40–5.13)**	<0.001
CMP	50	151	1.26 (0.91–1.73)	0.17
**CHF**	59	498	**3.54 (2.70–4.64)**	<0.001

a CAD, coronary artery disease; CHF, congestive heart failure; CI, confidence interval; CMP, cardiomyopathy; CREST, calcinosis, Raynaud's syndrome, esophageal dysmotility, sclerodactyly, and telangiectasia; EDS, Ehlers-Danlos syndrome; M, men; PAH, pulmonary arterial hypertension; PH, pulmonary hypertension; RR, relative risk, SLE, systemic lupus erythematosus; SSc, systemic sclerosis; yr, year.

b Relative risk assessed using Cox Model.

c Bold- increased risk in women ≥50 years of age.

**Table 14:** Relative risk of developing a comorbidity in men with Sjögren's disease that are ≥50 years of age (*n* = 1,880).

**Comorbidity**	** <50 yr^a^**	**≥50 yr**	**HR (±CI)**	***P*** **-value^b^**
Fibromyalgia	49	107	0.64 (0.46–0.90)	0.01
Depression	67	187	0.85 (0.65–1.13)	0.27
Pain	55	141	0.77 (0.56–1.05)	0.095
Migraine	8	14	0.51 (0.21–1.23)	0.13
Raynaud's syndrome	19	43	0.67 (0.39–1.16)	0.15
SSc	5	28	1.64 (0.63–4.25)	0.31
CREST	1	3	1.14 (0.11–11.49)	0.91
**Stroke^c^**	**8**	**67**	**2.65 (1.27–5.54)**	**0.009**
**PAH**	**6**	**74**	**3.76 (1.64–8.66)**	**0.002**
**PH**	**8**	**75**	**2.91 (1.40–6.03)**	**0.004**
EDS	2	0	0.00 (0.00–Inf)	1
Hypermobile syndrome	0	1	0.00 (0.00–Inf)	1
RA	26	126	1.52 (0.99–2.32)	0.054
SLE	16	45	0.84 (0.48–1.50)	0.56
Polymyositis	5	7	0.40 (0.13–1.26)	0.12
Dermatomyositis diag	3	2	0.18 (0.03–1.10)	0.064
Myocarditis	0	2	0.00 (0.00–Inf)	1
Lymphoma	12	31	0.76 (0.39–1.48)	0.41
**Atherosclerosis/CAD**	**28**	**372**	**4.56 (3.10–6.71)**	**<0.001**
**Myocardial infarction**	**7**	**91**	**4.14 (1.91–8.94)**	**<0.001**
CMP	9	40	1.46 (0.71–3.03)	0.3
**CHF**	**8**	**113**	**4.48 (2.18–9.19)**	**<0.001**

a CAD, coronary artery disease; CHF, congestive heart failure; CI, confidence interval; CMP, cardiomyopathy; CREST, calcinosis, Raynaud's syndrome, esophageal dysmotility, sclerodactyly, and telangiectasia; EDS, Ehlers-Danlos syndrome; M, men; PAH, pulmonary arterial hypertension; PH, pulmonary hypertension; RR, relative risk; SLE, systemic lupus erythematosus; SSc, systemic sclerosis.

b Relative risk assessed using Cox Model.

c Bold- increased risk in men ≥50 years of age.

## Discussion

A female to male bias has been reported for the rheumatic autoimmune disease's dermatomyositis (2:1), rheumatoid arthritis (3:1), SLE (7:1), SSc (12:1) and Sjögren's disease (6:1–14:1) (16, 17, 19–21). In this retrospective study of 13,849 patients with primary and/or secondary Sjögren's disease we found a sex ratio of 6.4:1 women to men which is somewhat lower than estimates from other large studies, although findings vary. Cardiovascular diseases also display strong sex differences with most heart diseases occurring more often in men like myocardial infarction, atherosclerosis/CAD, myocarditis, cardiomyopathy/dilated cardiomyopathy, and pulmonary hypertension while others occur more often in women, particularly after menopause, like hypertension, PAH, and stroke (24–28). In this study we found that men with Sjögren's disease had a greater risk of developing cardiovascular diseases than women (sex ratio, Table 1; Figure 1), while women had a greater risk of developing another rheumatic autoimmune disease and/or a chronic pain condition like EDS, migraine, hypermobile syndrome or fibromyalgia (Figure 1). At diagnosis, women were more likely to have fibromyalgia, pain and EDS (Table 8). EDS/hypermobile syndromes did not occur with high frequency in the record; however, it is a relatively common condition that occurs within the same demographic population of around 90% white women with a high percentage overlap with fibromyalgia (29), and historically has been under-diagnosed. Future research is needed to determine whether hypermobile EDS is an important comorbidity in Sjögren's disease.

Sjögren's disease is a chronic inflammatory condition where T and B cells directed against self-antigens from the exocrine glands lead to autoantibody and immune complex formation, tissue damage and inflammation (17). We found in this study that more women were positive for ANA and SSA/Ro autoantibodies compared to men (Table 3). We and others have hypothesized that increased inflammation, autoantibodies and immune complex deposition in women with autoimmune diseases increases the risk of developing other rheumatic autoimmune and cardiovascular diseases, especially after menopause (age 50) [reviewed in (17)] (17, 30, 31). Patients with Sjögren's disease have also been reported to have more hypertension and type II diabetes which increase the risk for cardiovascular disease (32), but to smoke less because of symptoms of dry mouth (30). Multiple studies have demonstrated an inverse correlation of smoking and Sjögren's disease or for smoking and focal lymphocytic sialadenitis (33–40), but differences between sex have not been reported. In this study a higher percentage of men with Sjögren's disease were smokers than women (49% men vs. 32% women), although the number of patients with data on smoking is relatively low (Table 2). Men are at an increased risk of developing cardiovascular disease compared to women (24–28) and the increased inflammation associated with Sjögren's disease would likely further promote the pathogenesis of cardiovascular disease in men.

Apart from the exocrine glands, patients with Sjögren's disease have extraglandular manifestations that affect their joints, lungs, kidneys, small vessels, as well as central and peripheral nervous system (4). Severe and chronic fatigue and pain are frequently reported by patients with Sjögren's disease and are associated with sleep disturbance and mood disorders (12, 41). Not only do these symptoms of Sjögren's disease overlap with fibromyalgia, which has been reported to occur in around 15–30% of patients with Sjögren's disease (6, 42), they also overlap with hypermobile EDS and hypermobile syndrome/hypermobile syndrome disorder (43–46). In this study we found that fibromyalgia occurred in 25% of patients with Sjögren's disease and was more frequent in women than men with Sjögren's disease (12:1), similar to other reports. Similarly, we found that more women with Sjögren's disease experienced depression (8:1), pain (8:1), migraine (23:1), EDS (22:1), and hypermobile syndrome (31:1) than men with Sjögren's disease. All of these conditions are known to occur more often in women than men (47–50). Our findings confirm known sex differences and provide an assessment of their frequency in a large cohort of patients with Sjögren's disease.

Our results show that women with Sjögren's disease are at a higher risk than men of having other rheumatic autoimmune diseases, depression, pain, migraine, fibromyalgia, EDS and hypermobile syndrome. However, we recognize that our study has certain limitations. A major limitation of the study is that as a retrospective study with a large number of patients we were not able to confirm whether the diagnosis of patients with Sjögren's disease was performed by a rheumatologist with expertise in this area. Likewise, we were not able to verify cases for such a large number of patients or distinguish primary from secondary Sjögren's disease. However, less research exists on secondary Sjögren's disease and so this data adds to that knowledge. Additionally, identifying patients using ICD-9/10 codes may over-represent the number of patients diagnosed with Sjögren's disease because the codes may be used to identify patients for work up but may not lead to a diagnosis. If patients that are included in the study do not have Sjögren's disease, this could affect the data leading to inaccurate conclusions. Even though this study included a large cohort of Sjögren's disease patients (13,849), analysis of 22 comorbidities by sex and age left small numbers of men for some comparisons and small numbers of patients for some comorbidities. If a higher number of patients were examined for those cases, the results may change. However, this study is the first to our knowledge to study comorbidities in Sjögren's disease by sex and age at diagnosis. Additionally, the lower risk in men with Sjögren's disease for comorbidities may be related to the far fewer number of men in the study. However, this study is the largest to date to our knowledge examining the largest number of comorbidities and with the greatest number of men available for analysis. The study may reflect trends observed in the general population and sex differences that exist in comorbidities in the general population may influence results, such as increased cardiovascular disease in men. Future studies should examine the EMR for a similar time-period to determine whether key comorbidities such as fibromyalgia and cardiovascular disease have the same sex difference or whether these conditions occur more frequently in men or women that have Sjögren's disease. This study is the first to our knowledge to examine sex differences for these 22 comorbidities in Sjögren's disease.

## Conclusions

The results of our study from patients at Mayo Clinic identified by ICD-9/10 codes showed that the top comorbidities in Sjögren's disease were fibromyalgia, depression, pain, and atherosclerosis. Women with Sjögren's disease were more likely to develop other rheumatic autoimmune diseases, fibromyalgia and experience pain, depression, migraine, EDS and hypermobile syndrome whereas men with Sjögren's disease were more likely to have cardiovascular disease and stroke. Future studies are needed to determine whether hypermobile EDS/hypermobile syndrome are important comorbidities in patients with Sjögren's disease.

### Perspectives and significance

This study reports, for the first time, data on 22 comorbidities that occur in Sjögren's disease according to sex and age at diagnosis and after diagnosis based on ICD-9/10 codes for Sjögren's disease from the Mayo Clinic medical record. Although it is known that rheumatic diseases occur more often in women and most cardiovascular diseases occur more often in men, this relationship has not been previously reported for these comorbidities in Sjögren's disease. Importantly, this study found that women with Sjögren's disease had an increased risk of developing chronic pain syndromes like fibromyalgia, migraine, depression, pain, hypermobile syndrome and Ehlers-Danlos syndrome. In contrast, men with Sjögren's disease were at an increased risk of cardiovascular disease. Our findings reveal that rheumatic autoimmune diseases, cardiovascular diseases and pain-related conditions present clinically at a similar time as Sjögren's disease (2.6 years after diagnosis), which suggests that sex hormone effects on the immune response may be important in determining the pathogenesis of disease in a sex-specific manner. Although it is well-known that sex hormones influence immunity to promote autoimmune and cardiovascular diseases, our data suggest that this mechanism may also be important for the development of pain-related conditions like fibromyalgia and hypermobile syndrome and the development of one or more comorbidities with Sjögren's disease add to the burden of disease in these patients.

## Data availability statement

The datasets presented in this article are not readily available because they contain identifiable information from the Mayo Clinic electronic medical record. Requests to access the datasets should be directed to fairweather.delisa@mayo.edu.

## Ethics statement

Research carried out in this study was in compliance with the Helsinki Declaration. The study was approved by the Mayo Clinic Institutional Review Board and receipt of a waiver of the need to consent subjects was obtained.

## Author contributions

KB and AM-L acquired, analyzed, interpreted the data, and wrote the first draft of the manuscript. GB assisted in writing and editing the manuscript. HS and DH analyzed and interpreted data and edited the manuscript. JP, JS, GS, RP, and SW analyzed data and assisted in writing and editing the manuscript. EB, TR, PA, and PD assisted with data analysis and edited the manuscript. SL interpreted data and edited the manuscript. LS designed the study, interpreted data and assisted with writing and editing the manuscript. DF designed the study, analyzed and interpreted the data, and wrote the manuscript. All authors read and approved the final manuscript.

## Funding

This work was supported by philanthropic support from the Ralph E. Pounds and Kathy Olesker Pounds Fund in Research Related to Headache to TR, the Ralph E. Pounds and Kathy Olesker Pounds Fund in Research Related to Chronic Pain to DF, the Mayo Clinic Florida Research Accelerator for Clinicians Engaged in Research to LS, National Institutes of Health grants R01 HL164520, R21 AI145356, R21 AI152318, and R21 AI154927 to DF, American Heart Association 20TPA35490415 to DF, and National Institutes of Health R21 AI163302 to KB.

## Conflict of interest

The authors declare that the research was conducted in the absence of any commercial or financial relationships that could be construed as a potential conflict of interest.

## Publisher's note

All claims expressed in this article are solely those of the authors and do not necessarily represent those of their affiliated organizations, or those of the publisher, the editors and the reviewers. Any product that may be evaluated in this article, or claim that may be made by its manufacturer, is not guaranteed or endorsed by the publisher.
