# Supplementary material for: Sex differences in comorbidities associated with Sjögren's disease
Source: Front Med (Lausanne). 2022 Aug 4;9:958670. doi: 10.3389/fmed.2022.958670 (PMC9387724; doi:10.3389/fmed.2022.958670)
Supplement: Supplementary file 1 [file Table_1.docx]

Sex differences in comorbidities associated with Sjögren’s disease

**Supplemental Tables**

Katelyn A. Bruno, PhD et al.

Corresponding Author:
DeLisa Fairweather, PhD, Mayo Clinic, 4500 San Pablo Road, Jacksonville, Florida 32224, Tel: 904-953-6740, Fax: 904-953-7117, Email: [Fairweather.DeLisa@mayo.edu](mailto:Fairweather.DeLisa@mayo.edu)

| **Supplemental Table 1.** Likelihood of having comorbidity by sex at Sjögren’s disease diagnosis (*n* =13,849) | | | | |
| --- | --- | --- | --- | --- |
|  | | | | |
| **Comorbidity** | **Women**  **(*n*=11,969)** | **Men**  **(*n* = 1,880)** | **Total**  **(*n* = 13,849)** | ***P* value***^a^* |
| **Fibromyalgia** |  |  |  | **< 0.001** |
| Missing (*n*) | 8,779 | 1,612 | 10,391 |  |
| No Dx*^b^* | 2,508 (78.6%) | 173 (64.6%) | 2,681 (77.5%) |  |
| Dx | 682 (21.4%) | 95 (35.4%) | 777 (22.5%) |  |
| Depression |  |  |  | 0.442 |
| Missing (*n*) | 9,364 | 1,545 | 10,909 |  |
| No Dx | 2,151 (82.6%) | 283 (84.5%) | 2,434 (82.8%) |  |
| Dx | 454 (17.4%) | 52 (15.5%) | 506 (17.2%) |  |
| Pain |  |  |  | 0.736 |
| Missing (*n*) | 9,956 | 1,618 | 11,574 |  |
| No Dx | 1,640 (81.5%) | 211 (80.5%) | 1,851 (81.4%) |  |
| Dx | 373 (18.5%) | 51 (19.5%) | 424 (18.6%) |  |
| Migraine |  |  |  | 0.149 |
| Missing (*n*) | 11,375 | 1,854 | 13,229 |  |
| No Dx | 548 (92.3%) | 22 (84.6%) | 570 (91.9%) |  |
| Dx | 46 (7.7%) | 4 (15.4%) | 50 (8.1%) |  |
| Raynaud’s |  |  |  | 0.409 |
| Missing (*n*) | 10,674 | 1,792 | 12,466 |  |
| No Dx | 1,131 (87.3%) | 74 (84.1%) | 1205 (87.1%) |  |
| Dx | 164 (12.7%) | 14 (15.9%) | 178 (12.9%) |  |
| SSC |  |  |  | 0.764 |
| Missing (*n*) | 11,427 | 1,839 | 13,266 |  |
| No Dx | 497 (91.7%) | 39 (95.1%) | 536 (91.9%) |  |
| Dx | 45 (8.3%) | 2 (4.9%) | 47 (8.1%) |  |
| CREST |  |  |  | 1.000 |
| Missing (*n*) | 11,852 | 1876 | 13,728 |  |
| No Dx | 113 (96.6%) | 4 (100%) | 117 (96.7%) |  |
| Dx | 4 (3.4%) | 0 (0%) | 4 (3.3%) |  |
| Stroke |  |  |  | 0.885 |
| Missing (*n*) | 11,546 | 1,784 | 13,330 |  |
| No Dx | 344 (81.3%) | 79 (82.3%) | 423 (81.5%) |  |
| Dx | 79 (18.7%) | 17 (17.7%) | 96 (18.5%) |  |
| PAH |  |  |  | 0.317 |
| Missing (*n*) | 11,157 | 1,771 | 12,928 |  |
| No Dx | 694 (85.5%) | 89 (81.7%) | 783 (85.0%) |  |
| Dx | 118 (14.5%) | 20 (18.3%) | 138 (15%) |  |
| PH |  |  |  | 0.189 |
| Missing (*n*) | 11,162 | 1766 | 12,928 |  |
| No Dx | 702 (87.0%) | 94 (82.5%) | 796 (86.4%) |  |
| Dx | 105 (13.0%) | 20 (17.5%) | 125 (13.6%) |  |
| EDS |  |  |  | 1.000 |
| Missing (*n*) | 11,904 | 1,877 | 13,781 |  |
| No Dx | 61 (93.8%) | 3 (100%) | 64 (94.1%) |  |
| Dx | 4 (6.2%) | 0 (0%) | 4 (5.9%) |  |
| Hypermobile |  |  |  | 0.290 |
| Missing (*n*) | 11,907 | 1,878 | 13,785 |  |
| No Dx | 53 (85.5%) | 1 (50%) | 54 (84.4%) |  |
| Dx | 9 (14.5%) | 1 (50%) | 10 (15.6%) |  |
| RA |  |  |  | 0.788 |
| Missing (*n*) | 10,324 | 1,697 | 12,021 |  |
| No Dx | 1494 (90.8%) | 165 (90.2%) | 1659 (90.8%) |  |
| Dx | 151 (9.2%) | 18 (9.8%) | 169 (9.2%) |  |
| SLE |  |  |  | 0.505 |
| Missing (*n*) | 10,852 | 1,795 | 12,647 |  |
| No Dx | 973 (87.1%) | 72 (84.7%) | 1045 (86.9%) |  |
| Dx | 144 (12.9%) | 13 (15.3%) | 157 (13.1%) |  |
| Polymyositis |  |  |  | 0.126 |
| Missing (*n*) | 11,857 | 1,866 | 13,723 |  |
| No Dx | 93 (83.0%) | 14 (100%) | 107 (84.9%) |  |
| Dx | 19 (17.0%) | 0 (0%) | 19 (15.1%) |  |
| Dermatomyositis |  |  |  | 0.611 |
| Missing (*n*) | 11,908 | 1,873 | 13,781 |  |
| No Dx | 50 (82.0%) | 5 (71.4%) | 55 (80.9%) |  |
| Dx | 11 (18.0%) | 2 (28.6%) | 13 (19.1%) |  |
| Myocarditis |  |  |  | 0.317 |
| Missing (*n*) | 11,943 | 1,875 | 13,818 |  |
| No Dx | 18 (69.2%) | 2 (40.0%) | 20 (64.5%) |  |
| Dx | 8 (30.8%) | 3 (60.0%) | 11 (35.5%) |  |
| Lymphoma |  |  |  | 0.184 |
| Missing (*n*) | 11,673 | 1,822 | 13,495 |  |
| No Dx | 248 (83.8%) | 44 (75.9%) | 292 (82.5%) |  |
| Dx | 48 (16.2%) | 14 (24.1%) | 62 (17.5%) |  |
| Atherosclerosis |  |  |  | 0.832 |
| Missing (*n*) | 10,464 | 1,375 | 11,839 |  |
| No Dx | 1270 (84.4%) | 424 (84.0%) | 1694 (84.3%) |  |
| Dx | 235 (15.6%) | 81 (16.0%) | 316 (15.7%) |  |
| Myocardial Infarction |  |  |  | 0.391 |
| Missing (*n*) | 11,612 | 1,751 | 13,363 |  |
| No Dx | 306 (85.7%) | 106 (82.2%) | 412 (84.8%) |  |
| Dx | 51 (14.3%) | 23 (17.8%) | 74 (15.2%) |  |
| Cardiomyopathy |  |  |  | 0.645 |
| Missing (*n*) | 11,738 | 1,821 | 13,559 |  |
| No Dx | 206 (89.2%) | 51 (86.4%) | 257 (88.6%) |  |
| Dx | 25 (10.8%) | 8 (13.6%) | 33 (11.4%) |  |
| CHF |  |  |  | 0.275 |
| Missing (*n*) | 11,280 | 1,718 | 12,998 |  |
| No Dx | 589 (85.5%) | 133 (82.1%) | 722 (84.8%) |  |
| Dx | 100 (14.5%) | 29 (17.9%) | 129 (15.2%) |  |

| *^a^* Fisher's Exact Test for Count Data  *^b^*Abbreviations: CAD, coronary artery disease; CHF, congestive heart failure; CMP, cardiomyopathy; CREST, calcinosis; Dx, diagnosis; Raynaud, oesophageal dysmotility, sclerodactyly, and telangiectasia; EDS, Ehlers-Danlos syndrome; M, men; PAH, pulmonary arterial hypertension; PH, pulmonary hypertension; SLE, systemic lupus erythematosus; SSc, systemic sclerosis; W, women. |
| --- |

**Supplementary Figure 2**. Average number of years (mean) until diagnosis of comorbidity in women and men after diagnosis of Sjögren’s disease

| **Comorbidity** | **Women (*n* = 11,969)** | **Men (*n* = 1,880)** | **Total (*n* = 13,849)** |
| --- | --- | --- | --- |
| **Fibromyalgia** |  |  |  |
| Missing | 8,779 | 1,612 | 10,391 |
| N | 3,190 | 268 | 3,458 |
| Mean*^a^* | 1.1 | 0.3 | 1.1 |
| Median (range) | 0.1 (-19.4, 35.0) | 0.0 (-17.1, 16.2) | 0.1 (-19.4, 35.0) |
|  |  |  |  |
| **Depression** |  |  |  |
| Missing | 9,364 | 1,545 | 10,909 |
| N | 2,605 | 335 | 2,940 |
| Mean | 2.5 | 2.9 | 2.5 |
| Median (range) | 0.9 (-19.1, 31.0) | 1.1 (-9.4, 19.6) | 0.9 (-19.1, 31.0) |
|  |  |  |  |
| **Pain** |  |  |  |
| Missing | 9,956 | 1,618 | 11,574 |
| N | 2,013 | 262 | 2,275 |
| Mean | 2.3 | 2.2 | 2.3 |
| Median (range) | 0.7 (-13.9, 20.8) | 0.7 (-8.8, 19.9) | 0.7 (-13.9, 20.8) |
|  |  |  |  |
| **Migraine** |  |  |  |
| Missing | 11,375 | 1,854 | 13,229 |
| N | 594 | 26 | 620 |
| Mean | 3.7 | 3.0 | 3.6 |
| Median (range) | 2.3 (-2.4, 22.6) | 1.3 (-2.1, 19.9) | 2.2 (-2.4, 22.6) |
|  |  |  |  |
| **Raynaud's** |  |  |  |
| Missing | 10,674 | 1,792 | 12,466 |
| N | 1,295 | 88 | 1,383 |
| Mean | 2.0 | 1.5 | 1.9 |
| Median (range) | 0.2 (-17.5, 23.0) | 0.1 (-11.0, 16.2) | 0.1 (-17.5, 23.0) |
|  |  |  |  |
| **Systemic Sclerosis** |  |  |  |
| Missing | 11,427 | 1,839 | 13,266 |
| N | 542 | 41 | 583 |
| Mean | 2.9 | 2.0 | 2.9 |
| Median (range) | 1.0 (-16.1, 20.8) | 0.5 (-1.4, 15.7) | 1.0 (-16.1, 20.8) |
|  |  |  |  |
| **CREST** |  |  |  |
| Missing | 11,852 | 1,876 | 13,728 |
| N | 117 | 4 | 121 |
| Mean | 5.0 | 6.7 | 5.0 |
| Median (range) | 3.1 (-1.3, 20.6) | 5.5 (0.0, 15.6) | 3.1 (-1.3, 20.6) |
|  |  |  |  |
| **Stroke** |  |  |  |
| Missing | 11,546 | 1,784 | 13,330 |
| N | 423 | 96 | 519 |
| Mean | 3.1 | 2.9 | 3.0 |
| Median (range) | 1.5 (-13.1, 20.4) | 1.4 (-10.0, 21.7) | 1.5 (-13.1, 21.7) |
|  |  |  |  |
| **PAH** |  |  |  |
| Missing | 11,157 | 1,771 | 12,928 |
| N | 812 | 109 | 921 |
| Mean | 3.2 | 1.8 | 3.1 |
| Median (range) | 1.4 (-13.6, 20.4) | 1.0 (-16.9, 17.2) | 1.3 (-16.9, 20.4) |
|  |  |  |  |
| **Pulmonary HTN** |  |  |  |
| Missing | 11,162 | 17,66 | 12,928 |
| N | 807 | 114 | 921 |
| Mean | 3.5 | 2.1 | 3.3 |
| Median (range) | 1.6 (-12.0, 20.4) | 1.2 (-16.9, 17.2) | 1.5 (-16.9, 20.4) |
|  |  |  |  |
| **EDS** |  |  |  |
| Missing | 11,904 | 1,877 | 13,781 |
| N | 65 | 3 | 68 |
| Mean | 1.1 | 4.9 | 1.3 |
| Median (range) | 0.2 (-14.2, 15.8) | 0.5 (0.0, 14.3) | 0.3 (-14.2, 15.8) |
|  |  |  |  |
| **Hypermobile syndrome** |  |  |  |
| Missing | 11,907 | 1,878 | 13,785 |
| N | 62 | 2 | 64 |
| Mean | 0.7 | 0.0 | 0.7 |
| Median (range) | 0.1 (-8.2, 15.8) | 0.0 (-0.0, 0.0) | 0.0 (-8.2, 15.8) |
|  |  |  |  |
| **Rheumatoid arthritis** |  |  |  |
| Missing | 10,324 | 1,697 | 12,021 |
| N | 1,645 | 183 | 1,828 |
| Mean | 3.3 | 3.1 | 3.3 |
| Median (range) | 1.1 (-15.9, 21.7) | 1.1 (-10.3, 19.4) | 1.1 (-15.9, 21.7) |
|  |  |  |  |
| **SLE** |  |  |  |
| Missing | 10,852 | 1,795 | 12,647 |
| N | 1,117 | 85 | 1,202 |
| Mean | 2.4 | 1.7 | 2.3 |
| Median (range) | 0.4 (-12.9, 20.8) | 0.2 (-8.7, 15.8) | 0.4 (-12.9, 20.8) |
|  |  |  |  |
| **Polymyositis** |  |  |  |
| Missing | 11,857 | 1,866 | 13,723 |
| N | 112 | 14 | 126 |
| Mean | 1.9 | 1.2 | 1.9 |
| Median (range) | 0.3 (-14.8, 31.4) | 0.1 (0.0, 5.4) | 0.3 (-14.8, 31.4) |
|  |  |  |  |
| **Dermatomyositis** |  |  |  |
| Missing | 11,908 | 1,873 | 13,781 |
| N | 61 | 7 | 68 |
| Mean | 2.4 | -1.4 | 2.0 |
| Median (range) | 0.9 (-5.2, 14.9) | 0.3 (-12.9, 3.5) | 0.9 (-12.9, 14.9) |
|  |  |  |  |
| **Myocarditis** |  |  |  |
| Missing | 11,943 | 1,875 | 13,818 |
| N | 26 | 5 | 31 |
| Mean | 0.4 | 0.7 | 0.4 |
| Median (range) | 0.0 (-9.1, 8.0) | -0.1 (-1.9, 6.8) | 0.0 (-9.1, 8.0) |
|  |  |  |  |
| **Lymphoma** |  |  |  |
| Missing | 11,673 | 1,822 | 13,495 |
| N | 296 | 58 | 354 |
| Mean | 3.6 | 1.9 | 3.4 |
| Median (range) | 1.8 (-11.3, 18.0) | 1.1 (-4.5, 15.6) | 1.6 (-11.3, 18.0) |
|  |  |  |  |
| **Atherosclerosis/CAD** |  |  |  |
| Missing | 10,464 | 1,375 | 11,839 |
| N | 1,505 | 505 | 2,010 |
| Mean | 3.5 | 3.2 | 3.5 |
| Median (range) | 2.1 (-20.0, 23.0) | 1.7 (-11.7, 21.7) | 2.0 (-20.0, 23.0) |
|  |  |  |  |
| **Myocardial Infarction** |  |  |  |
| Missing | 11,612 | 1,751 | 13,363 |
| N | 357 | 129 | 486 |
| Mean | 3.8 | 3.0 | 3.6 |
| Median (range) | 2.5 (-12.2, 23.0) | 1.5 (-15.0, 19.9) | 2.2 (-15.0, 23.0) |
|  |  |  |  |
| **Cardiomyopathy** |  |  |  |
| Missing | 11,738 | 1,821 | 13,559 |
| N | 231 | 59 | 290 |
| Mean | 4.2 | 4.5 | 4.2 |
| Median (range) | 2.4 (-10.1, 23.0) | 2.9 (-5.7, 19.7) | 2.6 (-10.1, 23.0) |
|  |  |  |  |
| **CHF** |  |  |  |
| Missing | 11,280 | 1,718 | 12,998 |
| N | 689 | 162 | 851 |
| Mean | 3.5 | 2.7 | 3.4 |
| Median (range) | 2.2 (-15.4, 20.1) | 1.7 (-12.4, 19.6) | 1.9 (-15.4, 20.1) |

*^a^* Calculated using Cox model.
